# Supplementary material for: Effect of emotional regulation on performance of shooters during competition: An ecological momentary assessment study
Source: PLoS One. 2025 Mar 25;20(3):e0318872. doi: 10.1371/journal.pone.0318872 (PMC11936280; doi:10.1371/journal.pone.0318872)
Supplement: Attachment — Submitted filename: Response to Reviewers.doc [file pone.0318872.s003.doc]

Reviewer #1

Response：We are very grateful for your comments regarding our manuscript “Effect of Emotional Regulation on Performance of Shooters during Competition: an Ecological Momentary Assessment”  All your suggestions are very important to us, both for composing the manuscript and our further research. We have studied comments carefully and have made corrections which we hope meet with approval.  Based on your advice, we amended the relevantsection in the manuscript. All your questions are answered below.

1. Consider revising the Abstract to more accurately reflect the key points and results of your research.
Response: Thank you for your insightful advisement. We have revised the statements regarding the key points and results in the abstract.
2. You refer to the flexibility in the usage of emotion regulation strategies. However, it is unclear which results of your study indicate high or low flexibility. Is it the sum score of the usage of all strategies? Conclusions regarding overall flexibility would require calculating a variable that numerically captures this construct. I am not sure if your data can provide information on emotional flexibility. Additionally, interpreting this in absolute terms would be difficult, as you would require a non-athlete control group to determine if your sample is indeed less flexible in their usage of emotional control strategies. Therefore, I suggest that you only interpret differences between your subgroups (gender and skill level). If you cannot operationalize the flexibility in emotional regulation, please avoid jumping to conclusions (e.g., “Athletes had low flexibility in emotion regulation …” [Abstract], “Low-level athletes have lower emotional regulation flexibility compared to high-level athletes, and they demonstrate inflexibility in both situational demands and specific” [4. Limitations], etc.).
Response: We are very grateful for your valuable suggestions. The calculation of emotion regulation flexibility is not about the total score of all strategies used. Emotion regulation flexibility is primarily reflected in the process by which individuals select appropriate matching strategies based on contextual demands (Naragon-Gainey et al., 2017; Martins et al., 2018; Sheppes & Meiran, 2008; Ortner & Pennekamp, 2020). Therefore, we have readjusted the calculation indicators of emotion regulation flexibility to make them more explicit. Previously, the calculation method separately computed and incorporated contextual demands and strategies into the model. Now, integrating the two into one indicator to reflect emotion regulation flexibility is more reasonable. We have also revised the limitations section regarding the comparison of emotion regulation flexibility among athletes of different skill levels.

3. You use some terminology from shooting sports that laypeople (such as myself) might find difficult to understand. Specifically, please elaborate on the outcomes of your models:
- What is the “proportion of good ten rings” [2.4 Analysis] exactly?
- What is meant by “speed of grip force” [Section 1.1] and how is it related to shooting performance?
- What exactly is meant by “slow the production of force” [Section 3.2] and again, how does this relate to performance?
Additionally, in Section 1.3 you refer to “rapidly changing competitive sports situations.” What do you mean by that? How is this related to shooting (i.e., what are the changing situations in a shooting competition)?
Response：Thank you very much for your invaluable suggestions, esteemed expert. I have incorporated explanations regarding the evaluation indicators of shooting performance into the corresponding sections of the text. Specifically, in Section 2.4, I have added introductions to the concepts and measurement methods of shooting ring value and the "proportion of good ten ring," as well as the data acquisition approaches. Additionally, based on a review of the original literature, I have further elaborated on "speed of grip force" in Section 1.1 and "slow the production of force" in Section 3.2. In Section 1.3, I have also included further clarifications of related concepts to rectify any previously unclear explanations. Your guidance has significantly enhanced the quality and clarity of the text.

4. Regarding your power analysis: If I am not mistaken, gPower is not able to perform power analyses for multilevel linear models. It appears that you computed a power analysis for a linear multiple regression with 4 predictors, resulting in the reported detectable effect sizes of f²=0.20 (I assume that you refer to the effect size parameter f², please make so explicitly). However, a power analysis based on a (non-hierarchical) linear regression is not suitable for a multilevel analysis.
Please conduct a suitable power analysis (for example, using a simulation-based approach) or use another method of justification for your sample size. You may find the following resource helpful:
https://online.ucpress.edu/collabra/article/8/1/33267/120491/Sample-Size-Justification https://shiny.ieis.tue.nl/sample_size_justification/
Response: Thank you very much for the resources and guidance you provided. We have carefully reviewed the relevant papers and instructional methods to correct our previous incorrect method for sample size calculation. Regarding the power analysis for MLM (multilevel modeling), we utilized the powerSim() function to simulate sample size calculation using the Monte Carlo method (Bolger & Laurenceau, 2013).

5. Please report how many surveys were completed and the extent of dropout you experienced. Additionally, specify if you had any exclusion criteria for the data, such as excluding participants with a survey completion rate below a certain threshold.
Response: Thank you for the reviewer's suggestions. We have incorporated the criteria for data exclusion in Section 2.1.

6. Please provide all survey questions asked in the 2.2 Measures section. The questions for the triggering event and the emotional intensity are missing.
Response: Thank you for the rigorous review, expert. We have supplemented the section on Contextual Demands with items related to triggering events and emotional intensity in the manuscript.

7. Please report the nationalities of the athletes.
Response: Additional clarification regarding the athletes' nationalities has been included in Section 2.1 of the manuscript.

8. The main effect models are interesting, but what about Level 1 interactions? At least two-way interactions might yield interesting results, especially in light of the literature, which has shown that engagement strategies are most effective in low-intensity emotional contexts, while disengagement strategies are more effective in high-intensity emotional contexts. You make several remarks regarding such an interaction:
- “Strategies that require more participation and effort, such as cognitive reappraisal, may be most effective in regulating low-intensity emotions, whereas strategies that involve disengagement from stimuli, such as distraction, may be most effective in regulating high-intensity emotions” [Section 1.3]-“In addition, engagement strategies are more effective at regulating low-intensity emotions, but require a greater allocation of cognitive resources” [Section 3.2]
Exploring these interactions in your models could provide a deeper understanding of how emotion regulation strategies function under varying emotional intensities. I would thus suggest to include a fourth model which encapsulates all two-way interactions of level 1 variables. I would thus suggest including a fourth model that encapsulates all two-way interactions of Level 1 variables. This additional analysis could provide a deeper understanding of how different emotion regulation strategies function under varying emotional intensities and contexts.
Response: Thank you very much for your detailed guidance. The interaction between situational demands and regulatory strategies has a significant impact on athletic performance, which is the focus of our research. It has been effectively concluded in previous studies that the engagement strategy is more effective under high situational demands, while the disengagement strategy is more effective under low situational demands. However, the impact of these interactive effects on athletic performance is the issue that this study is more concerned with. We have conducted a comprehensive discussion and repeated research, and made adjustments in the model part of the data analysis. Situational needs and emotional regulation strategies are considered as first-level variables, and different skill levels are considered as second-level variables. We analyze the impact of this interaction on athletic performance, while also testing whether this impact is consistent across groups of different athletic levels.

9. To strengthen the analysis, I suggest performing a model comparison analysis. Since Model 1 is not nested within Model 2, using an Information Criterion like the Akaike Information Criterion (AIC) and/or the Bayesian Information Criterion (BIC) would be appropriate. These criteria allow for the comparison of non-nested models by evaluating the trade-off between model fit and complexity.

Response: Thank you for your valuable suggestions, which have greatly supported my systematic understanding of the differences between report models. In future studies of this kind, I will complete my thesis report according to your advice. As we have currently overhauled the data analysis model, there is no comparative information between multiple models presented at the moment.

10. At the moment, you only report the random intercept. Could you also report the random effects of gender and (skill-)level?
Response: After reviewing the literature, we have changed our previous data analysis method. Considering that the article focuses on the relationship between situational demands and strategies in emotional regulation (two important processes of emotional regulation), we have integrated indicators of emotional regulation flexibility to more effectively reflect the dynamic relationship between emotional regulation and athletic performance. We have altered the analysis model used before, adding reports of random effects of skill level in the new analysis. Since previous studies have shown that gender only differs in emotional regulation strategies, considering that the research focus is on the flexibility of emotional regulation and shooting performance, which is more likely to be affected by different levels of athletic skill. Therefore, gender is included in the analysis as a control variable.

11. I noticed some inconsistencies in your descriptives, specifically in Table 1: The average ring value is approximately 10, or exactly 10.00 for male athletes. Isn’t this the highest possible value achievable, meaning that they shot the center of the target almost every time (I apologize, I am a layperson regarding shooting)? Is this correct? Could there be a ceiling effect in accuracy? Additionally, how does this align with the ten-ring ratio being approximately 35%? Shouldn’t this be close to 100% if the average ring value was 10? Please clarify these points and explain the potential discrepancy between the average ring value and the ten-ring ratio.
Response: Thank you for your valuable feedback. The scoring rings on the shooting target range from the outer 6-ring to the central 10-ring, with each whole number ring being divided into 10 parts (from 6.0 to 6.9, and so on, with the central 10-ring ranging from 10.0 to 10.9). The precise ring value recorded for the shooter includes a decimal point. A "good ten" usually refers to a score above 10.0. We have added this explanation in the section of the paper that measures shooting performance.

12. “National-level athletes have higher performance levels and significant differences in emotional experience and use of regulatory strategies, which may account for the significant differences in shooting performance.” [Section 3.1] This conclusion is likely confounded with athlete experience and habituation. National-level athletes have probably competed in many competitions and might have habituated to the emotional distress experienced during competition. Their experience might also be a reason for the better performance, raising the question of whether emotion regulation strategies are indeed the primary reason behind their greater success. Please discuss these results more carefully.
Response: Thank you for this valuable suggestion. We have revised some of the content in the discussion section.

13. The discussion of gender differences in Section 3.1 is somewhat brief. Please elaborate further on the differing results for females compared to males. What are the implications of these findings, and why do these differences exist?
Response: Thank you for your insightful question. Regarding gender differences, past research has primarily shown that they manifest in emotional regulation, such as women experiencing emotions more intensely and having a larger repertoire of emotional regulation strategies. However, in terms of overall emotional regulation capabilities, there is no significant gender difference. In our previous analysis, we did include gender as a variable in the model. But in our current revision of the paper, considering that the focus of the study is on the relationship between emotional regulation flexibility and athletic performance, we are treating gender as a control variable. Instead, we are exploring the impact of emotional regulation flexibility on athletic performance across athletes of varying skill levels to see if there are differences. Comparing this influence between genders seems unreasonable since there is no significant gender difference in emotional regulation capabilities and shooting performance. We have added this explanation in the section of the paper that discusses the measurement of shooting performance.

14. “However, the intensity and controllability of emotions are averaged, making it difficult to examine the variability and degree of adaptation of emotional regulation strategies under different situational demands.” [Section 4. Limitations] I do not understand this limitation. You used a hierarchical model for your analysis, so you did not average over intensity and controllability of emotions, correct?
Response: The original statement may lead to ambiguous interpretations. In terms of data analysis, there is no averaging of the intensity and controllability of emotions. Athletes assess emotional events and intensity at a fixed time each day, which can lead to a subjective perception that has been averaged, rather than reflecting the real-time emotional experiences and strategic choices during competitions. To clarify this point, we have revised the statement "However, athletes' retrospective assessment of emotional induction, intensity, and controllability is an evaluation of the overall situation over a short period, not the emotional intensity and controllability that occur in real-time during training and competitions. This also makes it difficult to examine the dynamic changes in athletes' emotional regulation strategies and situational demands over this period. This is an inherent limitation of survey research, which is the inability to record more detailed data more densely."

15. Please discuss the limitations in more detail. For instance, you relied solely on retrospective self-reported data. This requires athletes to a) remember their emotions and strategies thoroughly and b) be able to clearly identify their used strategy, a task that is arguably even hard for mental health practitioners. Also, you did not assess physiological data, which might be crucial to understand in light of the shooting performance. Additionally, the lack of preregistration is a significant limitation, as preregistration is considered part of good scientific practice. Please reflect on these and any other limitations in your study.
Response: Thank you very much for your insightful guidance. We acknowledge that our discussion in the Limitations section was not as comprehensive as it could have been. In line with your suggestions, we have now expanded upon the limitations related to data collection, research design, and other aspects of the research process.

16. A Conclusion section at the end of your discussion would round off the paper nicely.
Response: We appreciate your advice and have added a conclusion at the end, succinctly summarizing the significant findings of this study.

17. The manuscript would benefit from increased clarity in certain sections. For instance:
• “Expressive suppression is the most commonly used strategy by athletes in intense competition with limited time （Beatty & Janelle，2016）, but the effective time is short, and the negative experience caused by high intensity may disrupt the movement structure.” [Section 1.1] Could you please elaborate on the statement “and the negative experience caused by high intensity may disrupt the movement structure”? This phrase would benefit from further explanation to clarify its meaning and implications
• “The prerequisite for effective emotion regulation strategies is the context that triggers emotional changes.” [Section 1.1] Unfortunately, I do not understand what you mean. Could you clarify this?
• “Previous studies have focused on exploring regulation strategies or patterns that are applicable to sport situations, and compared to life situations, sport situations are constantly changing, and the applicability of specific regulation strategies is challenged.” [Section 1.1] What do you mean by “compared to life situations, sport situations are constantly changing”? It's debatable whether sport situations are inherently more dynamic than everyday life scenarios, which can also be highly variable and unpredictable. Could you please elaborate on this comparison and provide evidence or specific examples to support your assertion?
• “The ability-based model suggests that emotional clarity, emotional tolerance, and emotional regulation flexibility are the potential for individuals to regulate emotions.” [Section 1.2] Please clarify what you mean by “are the potential for individuals to regulate emotions”
• “Rumination (disengagement with a tendency to avoid difficulties) is also classified as a disengagement strategy （Goodman，2021)”I don’t understand what is meant by “disengagement with a tendency to avoid difficulties”, please clarify. [Section 1.2]
• “However, in rapidly changing competitive sports situations, the effects of specific emotion regulation strategies are not stable, and athletes need to flexibly choose and use different strategies based on changes in the situation to achieve good results.” [Section 1.3] What do you mean by “rapidly changing competitive sports situations”. How does this specifically relate to shooting?
• “…the second-level variables were individual differences among participants (level, gender)” At this point in the manuscript, it was unclear to me, what “level” means. Maybe re-label it to “skill-level” or something more comprehendible.
• “Model 2: A regression model with mean as the outcome variable is used to conduct […]” [Section 3.2] Please clarify which mean (of what?) is used as an outcome
Response: We are very grateful for the expert's meticulous and rigorous review, which plays a crucial role in enhancing the scientific rigor and comprehensibility of the article. In Section 1.1, we have further elaborated on the explanation of "the negative experience caused by high intensity may disrupt the movement structure." We continue to clarify and explain that "The prerequisite for effective emotion regulation strategies is the context that triggers emotional changes." "The ability-based model suggests that emotional clarity, emotional tolerance, and emotional regulation flexibility are the potential for individuals to regulate emotions." [Section 1.2] We have added corresponding discussions to further illustrate the perspective of the ability model. We continue to clarify "disengagement with a tendency to avoid difficulties" [Section 1.2], and "rapidly changing competitive sports situations" [Section 1.3]. In the process of competitive sports, there is a plethora of situational information (opponent interference, rapid score changes, unexpected factors), all of which are characterized by rapid changes and can potentially induce emotional fluctuations in athletes. Athletic information has a significant impact on athletes' performance on the field, and the most significant situational change in shooting sports comes from score fluctuations. "…the second-level variables were individual differences among participants (level, gender)" At this point in the manuscript, it was unclear to me what "level" means. We have standardized the use of "athletic level" throughout the text to replace the previous unclear reference, indicating the level of athletic skill that athletes achieve through competition. We have checked other parts of the text for unclear expressions, made uniform revisions, and marked them in different colored fonts.

18. Your manuscript sometimes uses inconsistent terminology which impairs flow and understandability:
• You define “the proportion of good ten rings” [2.4 Analysis] as one of the outcomes. However, you refer to this outcome as “Ten-ring Rations” [Table 1 to Table 3], or “proportion of perfect scores” [Section 3.2].
• You refer to one type of emotion regulation strategies as “engagement strategies” but you refer to this as “participation strategy” in Section 3.2
Response: Thank you for your suggestions on improving the paper. We have made uniform revisions to the inconsistencies in the text. It is very important to maintain consistent terminology throughout the research paper. We have reviewed the entire document and made the necessary changes.

19. Some statements in your manuscript require literature references:

Response: We have carefully reviewed each instance of literature citation and have supplemented those areas where references were missing. We have reviewed the textual expressions throughout the document, including those you pointed out and other areas that required additional literature citations. We have also checked and corrected any errors in citation formats within the entire text.

Thank you again for the detailed comments on this paper; I have gained a lot from them.

Reviewer #2:

Response：We are very grateful for your comments regarding our manuscript “Effect of Emotional Regulation on Performance of Shooters during Competition: an Ecological Momentary Assessment”  All your suggestions are very important to us, both for composing the manuscript and our further research. We have studied comments carefully and have made corrections which wehope meet with approval. Based on your advice, we amended the relevantsection in the manuscript. All your questions are answered below.

1. The authors mentioned that they used statistical software for data processing. However, the explanation lacked detail, such as which menus or features were utilized. This level of detail was necessary to allow other researchers to replicate the study described in this manuscript. As a suggestion, researchers could consider using statistical analysis with programming languages to facilitate easier replication.

Response：Response: Thank you for the important feedback, expert. We have provided a more detailed description of the data processing steps in Section 2.4. The data analysis was conducted using R version 3.6.1, which is a programming language. The specific statistical analysis and the dataset have been uploaded together to the data management platform.

2. In the manuscript, the authors mentioned the term 'ACC' but never explained its meaning. This could make it difficult for general readers to understand its significance.

Response: Thank you for your valuable feedback. We have added the full expansion of this acronym in the text and will use the full name throughout the manuscript.

3. The authors developed three regression models. In the first model, a two-level approach was used. In the second model, no predictors were included at the second level. Why did the authors choose not to use a standard multiple regression model? It appears there is no significant difference. Please explain.

Response: We are deeply grateful for your significant insights. The data in this study are longitudinal and we employed multilevel regression modeling to examine the impact of variables at different levels on the dependent variable. Taking into account the feedback from several reviewers, we have conducted a fresh statistical analysis of the data, focusing particularly on the influence of emotional regulation factors on athletic performance over time.

4. In the second model, the authors coded gender as 1 for males and 2 for females. Why did not the authors use 0 for males and 1 for females, given that gender was nominal and not ordinal? Please explain.

Response: We appreciate the rigorous review by the expert. We have corrected this issue in the paper and conducted the model testing again.

5. The researchers should consider providing the equation form of all the models generated, including the value of each coefficient and intercept. This will allow readers to visually assess the influence of each predictor on the target.
Response: Thank you for the expert's feedback. By consulting other similar research literature, we have supplemented the corresponding content in the data analysis table.

Reviewer #3:

Response: We are very grateful for your comments on our manuscript titled "Effect of Emotional Regulation on Performance of Shooters during Competition: an Ecological Momentary Assessment." We appreciate your recognition of the introduction section of this manuscript, and we are equally thankful for your insights on the research methods section, which have been very enlightening for our further revisions of the paper. Below, we will provide a detailed response to each of your questions and the revisions we have made accordingly.


1. Method
- The problem of the origin of the items:
- The authors point out that they largely chose items from other questionnaires to measure emotion regulation strategies, e.g. for cognitive reappraisal the item chosen was: ‘I tried to think about the situation differently.’ from the ‘reappraisal subscale’ of the Emotion Regulation Questionnaire (ERQ) by Gross, John (2003). The item ‘I made sure not to express what I was feeling’ according to the authors was selected from the same questionnaire (ERQ) to determine the Expressive suppression strategy. The ERQ questionnaire is very well known. Although the authors' items are somewhat related in content to the items in the questionnaire, I did not find items in the ERQ with the same content as presented by the authors (see Gross, John 2003, Table 2 - 1-reappraisal factor, 2-suppression factor).

- The authors indicate that ‘Acceptance was measured with an item derived from acceptance and mindfulness measures (Baer et al., 2004), “I tried to notice my thoughts and feelings without thinking of them as good or bad”. In the questionnaire in the source given, I also did not find an item with such content as indicated by the authors (see Baer et al., 2004 - Table 1 - Kentucky Inventory of Mindfulness Skills). There is instead a item such as: ‘I make judgments about whether my thoughts are good or bad’.

- The authors also indicate that ‘Rumination was measured with an item derived from rumination measures (Treynor et al., 2003), “I kept thinking about and dwelling on my anxiety.”. I did not find such an item in the scale provided in the literature either (see Treynor et al., 2003 - Table 1 - Ruminative Responses Scale).

It is therefore difficult to know where the items presented come from, and if they are related to the content of items from other questionnaires, what modifications they have undergone.
Response: We greatly appreciate your feedback, which is of significant importance for us to think more cautiously about this research. The measurement items used in this study are not the first time they have been employed in this way. The cognition appraisal item is not a question from the "reappraisal subscale" of the ERQ. We have fully drawn on research in the field of mental health (Goodman et al.'s 2021). We have also reviewed other literature and found that in daily tracking studies on emotion regulation, this type of assessment is also used, often selecting the most core content of the reappraisal subscale (from ERQ), such as "I tried to think about the situation differently." The use of expressive suppression is similarly approached, as seen in Catterson et al.'s research ("An Experience Sampling Approach to Emotion Regulation: Situational Suppression Use and Social Hierarchy"). The strategy of acceptance is also a description of the characteristic of "coping with negative mood that involves self-focused attention" (Treynor et al., 2003). Furthermore, we would like to clarify that there are many emotion regulation strategies, and individuals use these strategies in specific contexts. An individual's specific approach to dealing with emotions, as long as it aligns with the typical characteristics of a certain emotion regulation strategy, is often considered as the use of that strategy. In this study, we are more interested in tracking which types of strategies athletes choose and use in response to daily situational changes, rather than examining the use of emotion regulation strategies in terms of individual differences.

2. methodological problem:

Authors used single items with unknown validity and reliability to measure emotion regulation strategies. Even if we assume that a single item was selected from a validated and standardised questionnaire, the validity and reliability of a single item is unknown. Short versions of questionnaires are often used, but these require full re-validation (regardless of the original version). On the other hand, shortening a subscale of a questionnaire to a single item, which in addition has been chosen arbitrarily, is an oversimplification. Unfortunately, a scale consisting of a single item is often too short to be reliable, and the use of a single item is particularly discouraged when measuring such broad constructs as personality or emotional processes. The authors further pointed out, for example, that a specific question was used to measure emotional intensity. When it comes to emotional process, it is very difficult to even assess the reliability of such a single-question scale (test-retest), as this phenomenon is very dynamic. To measure emotion regulation strategies, I would suggest using a short, but recognised, standardised and validated questionnaire or constructing and validating your own questionnaire where the subscale consists of more than one item.

- The authors further write: ‘These strategies include cognitive appraisal, acceptance, problem solving, emotional suppression, rumination, and avoidance. The first three are categorised as engagement strategies, while the latter three are categorised as disengagement strategies'. - The authors selected some items and created new subscales from them as they saw fit. The authors then also analysed the results in the proposed subscales. This approach is also methodologically questionable. Combining selected questions into new subscales or questionnaires would have required theoretical analysis and psychometric re-validation, which unfortunately the authors did not do. In order to create new subscales from the questions used, it would have been necessary to first provide a theoretical perspective on the rationale for their creation, and then perform a psychometric analysis to verify the validity of the approach used.
Response: Thank you for your question regarding the research methodology. The six emotion regulation strategies used in the study were categorized into two groups based on meta-analytical work that has identified these six distinct and commonly employed strategies: three engagement strategies, which include cognitive reappraisal, acceptance, and problem-solving, and three disengagement strategies, which consist of rumination, expressive suppression, and avoidance (Aldao et al., 2010; Naragon-Gainey et al., 2017). This classification is also reflected in previous research (Goodman et al., 2021. How do people with social anxiety disorder manage daily stressors? Deconstructing emotion regulation flexibility in daily life).

3. Some questions need to be more explicit, e.g., the item on intensity of emotion would require clarification of what emotion is meant, e.g., positive, negative, etc.
Response: We greatly appreciate your valuable feedback. We have added an explanation regarding the emotional valence evoked by emotional events in both the measurement and results sections. The study primarily focuses on anxiety and stressful events experienced by athletes before and during competitions, which are predominantly negative emotions.

4. The authors further indicate” The number of participants required to compute the multilevel linear model with four predictor variables and two-tailed tests using Gpower3.1 was over 42, which provides sufficient power (i.e., greater than .80) to detect effect sizes ≥ .20.” Unfortunately, there are many more than 4 predictors in the model presented. In the final model presented in Table 3, there are as many as 8 expressions for fixed effects, and even before interaction effects, single predictors (without interaction) should still be included, resulting in a much larger number of predictors. Besides, the sample size should be determined for a specific effect size, among other things. It is not known what the name of the effect proposed in the paper is, and hence, among other things, it is difficult to assess its strength so as to determine the sample size. On another note, it is not known how the sample size was determined for such advanced models taking into account both fixed and random effects using Gpower3.1 software.
Response: Thank you very much for the resources and guidance you provided. We have carefully reviewed the relevant papers and instructional methods to correct our previous incorrect method for sample size calculation. Regarding the power analysis for MLM (multilevel modeling), we utilized the powerSim() function to simulate sample size calculation using the Monte Carlo method (Bolger & Laurenceau, 2013).

5. Results
- The authors indicate that: “Using ecological momentary assessment, we followed 57 athletes during competitions for a period of 6 days before the competition and 2 days during the competition” and then write that a multilevel linear model was used to analyze the longitudinal data. The main purpose of longitudinal research and analysis is to analyze changes over time in relation to various variables. Unfortunately, the authors did not model the time factor, and the data was analyzed as if it had been collected at one time. So I suggest including time factor as a fundamental element in the analysis of longitudinal studies. A good source for longitudinal analysis in multilevel modeling is a widely available textbook: Heck, R. H., Thomas, S. L., & Tabata, L. N. (2013). Multilevel and longitudinal modeling with IBM SPSS. Routledge.
Response: We are grateful for the esteemed reviewer's valuable comments. We have carefully reviewed the resources you provided to us and have modified our statistical model accordingly. The data for this study are longitudinal tracking data from an athlete population. In response to this suggestion, we have conducted a reanalysis of the statistical data. We have modeled the time factor and analyzed the impact of emotional regulation factors on athletic performance over time. This reanalysis focuses on how emotional regulation and shooting performance change in relation to each other as time progresses. The analysis aims to explore whether the influence of emotional regulation flexibility on athletic performance varies among athletes of different skill levels.
